# Potentiality of biodiesel and bioethanol production from feedstock in Bangladesh: A review

**DOI:** 10.1016/j.heliyon.2022.e11213

**Published:** 2022-10-27

**Authors:** Dalim Kumer Roy, Mohammad Zoynal Abedin

**Affiliations:** Department of Mechanical Engineering, Dhaka University of Engineering & Technology, Gazipur 1707, Bangladesh

**Keywords:** Biofuel, Biodiesel, Bioethanol, Edible, Non-edible, Agricultural residues, Agricultural products

## Abstract

The aim of this paper is to provide a review of the most promising opportunities for sustainable biofuel generations in Bangladesh. Many researchers provide their opinions with their experimental results, but there has been no overall statistics and potentiality for the sustainable production of biofuel such as bioethanol and biodiesel in Bangladesh. The paper reviews the recent statistical conditions and the potential of biodiesel and bioethanol production in Bangladesh compared to other countries. Basically, the paper focuses on the potentiality of various biofuel feedstocks like as soybean oil, mustard oil, cottonseed oil, sesame oil, coconut oil, algae, rubber seed oil, jatropha, karanja oil, castor, bahera, neem, rice bran oil, pitraj and also different types of residues, crops, fruits, wastes. Among these reviewed papers, it is revealed that Bangladesh can generate annually about 0.16 million tons of edible oil. In addition, Bangladesh has the ability to produce about 1001881 tons of biodiesel from 2387500 tons of non-edible oil. Also, 0.04 million metric tons of biodiesel can be made from rubber seed oil. On the other hand, about 32 metric tons of bioethanol from 65.36 metric tons of agricultural crop residues, and about 143670082.36 gallons of bioethanol from 10.22 million metric tons of potato that is enough to meet the demand of 5% bioethanol blend annually. Furthermore, Bangladesh can produce 31.65 million metric tons of bioethanol from rice residue and 1.34 million metric tons of crude rice bran oil from rice husk of the paddy. It is conjectured that these annual production of various feedstocks can be used as major sources of biofuel and also can meet the demands of biofuel in Bangladesh.

## Highlights

• Bangladesh has the ability to produce a sufficient amount of 1G and 2G biofuels from edible and non-edible oil feedstocks.
• Great potentiality of biodiesel production from the non-edible oils in Bangladesh.
• Bangladesh has great feasibility of large-scale bioethanol production from crops, residues, and fruits.
• Possibility of the reduction of greenhouse gas emission.
• Annual production of feedstocks is a major source of biofuel that meet the demands in Bangladesh.

## Graphical abstract

**Figure:**
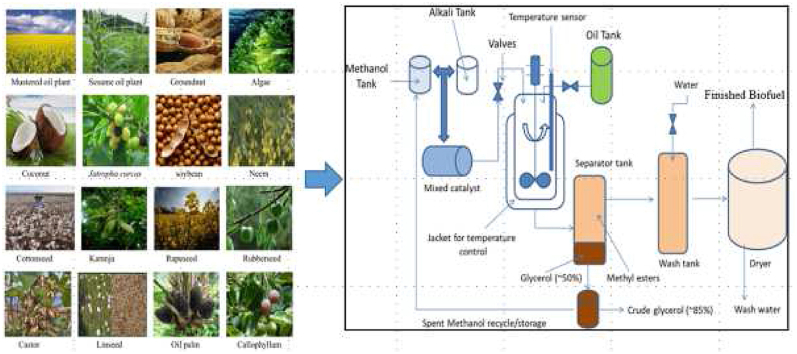

## 1 Introduction

### 1.1 Biofuels

A biofuel is one kind of renewable fuel that is made from biomass through name the geological processes and biological processes such as agriculture, prehistoric biological matter, and anaerobic digestion. In other words, biofuels can be created directly from living plants, or indirectly from wastes, agricultural product, agricultural residues [1, 2]. It is an environmentally-friendly fuel that is used as an alternative to fossil fuels. Biofuels can be liquid forms like biodiesel, green diesel, bioethanol, biobutanol, biomethanol, bio-oil [3]. Biofuels can also include gaseous or solid form such as syngas, biogas and wood pellets. The most common biofuel are biodiesel and ethanol where as biodiesel is derived from vegetable oils or animal fats, and bioethanol is produced by fermentation, especially from carbohydrates made in starch crops or sugar in the form of alcohol. The worldwide population is projected to reach 9800 million by 2050 and 12,000 million by 2100 [4]. With increasing population, the energy demand and/or requirement also increases in coming years [5, 6]. So it is essential to grow the biofuel production capacity in the world.

### 1.2 Primary energy status in Bangladesh

The total primary energy consumption of Bangladesh in 2001 was 141 lakh tons and in 2011 was 243 lakh tons including trade fuels and renewable resources [7]. The leading energy resources of Bangladesh are biomass and commercial energy resources. Commercial energy resources highly depend on natural gas, which is made locally and supply 53.61% energy of total energy used, whereas biofuel and waste, coal, crude oil, oil products, and hydropower are contributing 28.38%, 2.94%, 4.03%, 10.83%, and 0.2%, respectively in Bangladesh [8, 9]. But, after some decades natural gas and coal resources will eventually be exhausted all over the country as perused. So, the commercial energy resources of Bangladesh will be limited, and the need to increase imports of fuel from other countries every year. That will impact the country's economic condition. In this situation, only renewable energy such as biodiesel and bioethanol can meet the energy demand. That will help to move forward and improve economically and environmentally. In the fiscal year 2006, the total amount of gas supplied was 12344 kilotons of oil equivalen (ktoe), where it was 16,614 ktoe in 2011 [9]. Table 1 shows the total primary energy supplies in Terajoule for different years in Bangladesh.

**Table 1:** Total primary energy supplies for different years in Bangladesh [10].

Year	Coal (TJ)	Natural gas (TJ)	Hydro (TJ)	Biofuels and waste (TJ)	Oil (TJ)	Wind, solar (TJ)
1990	11781	156049	3182	287395	75533	
1995	13,434	230,015	1339	302,220	114,135	
2000	13,811	309,020	2761	319,391	130,781	
2005	17,682	452,583	2761	345,409	149,351	
2010	33,942	696,221	2779	356,687	158,001	
2015	95,050	884,880	2160	351,714	210,377	778
2019	165,740	10,68,016	2767	331,716	238,913	1353

### 1.3 Global views of biofuel

Global biofuel generation reached 105000 million liters in 2010, which was 17% higher than 2009, and biofuels supplies 2.7% of the total fuels for transportation in the world. In 2010, worldwide ethanol fuel generation reached 86,000 million liters. United States and Brazil are the two largest producer countries in the world and they supplies 90% of world production. On the other hand, European Union produced the largest amount of biodiesel in the world and they supplies 53% of global production in 2010 [1]. In 2018, United States and Brazil produced 69% of biofuels from global production, where European Union (EU-28) has generated 9% [11, 12]. The worldwide biodiesel, bioethanol, and hydrotreated vegetable oil (HVO) production increasing trend that will reach approximately 25% by 2024 [13,14].

The government of Bangladesh are allowing 5% ethanol to blend with octane fuel or gasoline. The governments studied that providing a 5% ethanol blend would needed 18 million liters in every year, which could be generated from 60,000 metric tons of broken rice per annum, and is about 3% of annual rice production, or approximately 62,000 tons of corn per annum that's less than 3% of annual corn production or about 97,000 tons of molasses production without the impact of food security [15]. The waste derived and waste minimization is the major concern of biofuel production and reduced emission for energy development [16, 17, 18, 19, 20, 21, 22].

## 2 Types of biofuel

### 2.1 First generation biofuels

First-generation or conventional biofuels are generated from sources like as vegetable oil, starch, or sugar. Some of the exoteric conventional biofuels are biodiesel fuel, bioethanol fuel, green diesel, bio ethers (also known as fuel ethers or oxygenated fuels, biogas, syngas (gasification), solid biofuels, vegetable oil fuel, and biofuel gasoline.

### 2.2 Second generation biofuels

Second generation or advanced biofuels are one kind of fuels that can be made from different types of biomass. A series of physical and chemical treatments might be required to convert lignocellulose biomass to liquid fuels suitable for transportation. Some of the exoteric advanced biofuels are lignocellulosic bioethanol, hydrotreating oil, FT oil, butanol, mixed alcohols and bio oil.

### 2.3 Third generation (sustainable) biofuels

Third generation biofuels are obtained from feedstock with better sustainability properties than second-generation biofuels. Currently, the most promising feedstock comes from microalgae.

## 3 Biodiesel

**Figure 1:**
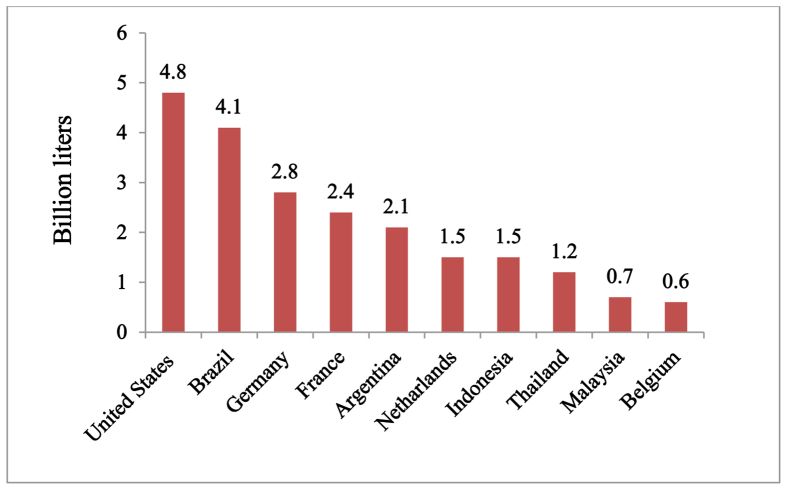
Biggest biodiesel production countries in the world in 2015 [23,24].

Biodiesel is used as an alternative fuel for diesel fossil fuel. Many countries are producing biodiesel in the world [23]. Figure 1 demonstrates the top most biodiesel producing countries in the world according to their production volume in 2015 [23,24]. The United States is the biggest biodiesel manufacturer country in the world. In 2014, United States produced 4700 million liters of biodiesel. But in 2015, Biodiesel production was 4800 million liters. The total production capacity is 7950 million liters that comes from 94 biodiesel plants. Feedstock in United States for biodiesel production is Soybean, Canola, Corn, animal fats. Indonesia is one of the largest biodiesel manufacturers in Asia with a total generation of 1500 million liters. The most significant feedstock of biodiesel generation in Indonesia is Palm [23].

Biodiesel is derived from vegetable oils or alcohol (methanol or ethanol) and animal fats by esterification, in the presence of an enzyme catalyst, acid or basic, and is also executed from transesterification. It is also known as fatty acid methyl ester (FAME), or fatty acid ethyl ester (FAEE) [25]. Nowadays, first-generation biodiesel is generated on a large scale and blended with fossil-derived diesel. It confirms the standards EN 14214 and ASTM D6751. Compared to fossil diesel, biodiesel has high biodegradability, low sulphur content, low stability (addition of antioxidants, which can be increased) [26], and high NOx (oxides of nitrogen) emissions when burned [27].

### 3.1 Sustainable biofuel from edible and non-edible sources

In Bangladesh most of the feedstock comes from edible and non-edible plants these can be used as a great source for biodiesel generation [23]. Table 2 contains three categories of feedstock that most of the sources are growing in Bangladesh [28]. Edible is food material and cannot fulfill the demand of biodiesel fuels. Annually requirements of edible oil are 0.5 million tons, whereas production capacity of edible about 0.16 million tons [9]. On the other hand, non edible oils can meet the demand of annual biodiesel production. Bangladesh has about 47,750 km length of road and rail side arid lands that can be used for the cultivating purposes of non-edible seeds. By using this amount of land in Bangladesh can produce 2387,500 tons of non-edible oil seeds annually, which will supply 10,01,881 tones of biodiesel on the basis of their oil content and yield [29]. Apart from, third-generation biofuel materials such as animal fats, microalgae, and fish oil, poultry fat oil, which are used as a great sources for biodiesel production.

**Table 2:** Feedstocks for biodiesel production [28].

Edible oil (First generation)	Non-edible oil (Second generation)	Other sources (Third generation)
Rapeseed oil	Jojoba	*Dunaliella salina* algae
Walnut oil	*Jatropha curcus*	*Chlorella vulgaris* algae
Sun flower oil	Mahuaindica	*Botryococcus braunii*
Soybean oil	Rubber seed	Waste cooking oil
Hazelnut oil	Neem	Animal Tallow oil
Rice bran oil	Nicotianatabacum	Chicken fat oil
Cotton seed oil	*Aleutites fordii*	Poultry fat oil
Tigernut oil	*Crambe abyssinica*	Biomass Pyrolysis oil
Raddish oil	*Sapindus mukorossi*	Fish oil
Palm oil	*Cerbera odollam*	
Cashewnut oil	Karanja	
Pistachio oil	*Cerbera odollam*	
Castor oil	Petroleum nut	
Mustard oil	Babassu tree	
	Milk bush	
	Silk cotton tree	
	Tall oil	

### 3.2 Potentiality of various (edible oil and non-edible oil) biodiesel feedstocks in Bangladesh

Bangladesh can produce a huge amount of biodiesel by utilizing both edible oil and non-edible oil sources such as Soybean oil (Glycine max), Mustard oil (*Brassica nigra*), Cottonseed oil, Sesame oil (*Sesamum indicum*), Mosna oil, Coconut oil, Algae, Rubber seed oil (RSO), Jatropha (*Jatropha curca*s), Karanja oil, Castor (*Ricinus communis*), Bahera (*T. bellirica*) Neem (*Azadirachta indica*), Rice bran oil (RBO), Pitraj (Aphanamixis Polystachya). These are described below-.

#### 3.2.1 Soybean oil

Bangladesh has 7–8 lakh hectares of land available for producing soybean oil and easily can meet 40% of its oil demand. Annually 17–18 lakh metric tons of Soybean oil could be produced [30]. Soybean produced per hectare about 2.25–2.4 metric tons [31]. Soybean contains about 20% oil, whereas only approximately 1%–2% of oil contains of all other pulse crops like as khesari, mung bean [32].

#### 3.2.2 Mustard oil

Cultivation of mustard oil is suitable in Bangladesh. Production possibility of Mustard oil is up to 22 lakh metric tons per annum [33]. By using the surplus Mustard oil can meet the demand of biodiesel [23]. Mustard oilseeds can be a great source of biodiesel fuel, and it compares with the standards ASTM or other diesel fuels [9]. Experimental results shows that the calorific value of mustard biodiesel fuel is 39.51 MJ/kg, which is same to the conventional fossil fuel, but the generation cost is slightly higher [34].

#### 3.2.3 Cottonseed oil

Cottonseed oil is derived from cotton seeds by removing cotton lint. It is one kind of vegetable oil, but it is not suitable as for human health [35]. Cotton trees can grow everywhere in Bangladesh and contains high amount of pesticide residue and fat. Cottonseeds produce approximately 77% Biodiesel with 20% Methanol [23, 36]. Compared to cottonseed biodiesel has a calorific value of 38.51 MJ/kg and kinematic viscosity of 7.2 m^2^/s at 40 1C, which is closed to other conventional diesel fuel [35].

#### 3.2.4 Sesame oil

Sesame is grown in almost everywhere in Bangladesh. In Bangladesh, 96000 hectares of available land for sesame oil cultivation and which supply 25000 metric tons of sesame biodiesel [37]. Sesame contains 42%–50% oil, 16%–18% carbohydrate, and 25% protein [38].

#### 3.2.5 Mosna oil

Mosna oil is one kind of edible oil sources that are grown mainly in Chittagong, Comilla, and Barisal [9]. The generation cost per liter of biodiesel from Mosna oil is Tk. 285 [35].

#### 3.2.6 Coconut oil

Coconut is a great feedstock for biodiesel production in Bangladesh. Coconut oil is derived from the kernel of mature coconuts by harvesting coconut palm. In 2004 to 2005, Bangladesh produced about 907255 metric tons of coconut by utilizing 12825 acres of land [39]. Southern part of the country is well suited for cultivating Coconut like as St. Martin's in cox's bazaar [9, 33]. Coconut contain high amount of bio oil, then Soybean and Mustard. The properties of coconut oil compared to biodiesel satisfied with ASTM standards [40].

#### 3.2.7 Rubber seed oil (RSO)

Bangladesh has approximately 91.8 thousand hectares of land which can be used for cultivating rubber seeds [41]. Annual production capacity of rubber seed oil per hectare is 217 kg oil/ha [42]. Bangladesh can produce 0.04 million MT of biodiesel from rubber seed oil in every year [43]. Rubber seeds yield 49% oil [44].

#### 3.2.8 Jatropha oil

*Jatropha curcas* can be a great sources of second generation biofuels in Bangladesh to fulfill future energy demand. It is also known as Jamalgota, Arenda, Verenda, Chanda. The oil content of *Jatropha curcas* is about 30–45% [45]. As the free fatty acids (FFA) of Jatropha oil is 14.02% [46]. Ethanol extractions from jatropha husk and jatropha stem are 0.14 L/kg and 0.20 L/kg, respectively [47]. The estimated amount of biodiesel production from *Jatropha curcas* is 1.92 tons per hectare per annum and the prospect of biodiesel production is 0.62 million metric tons per annum [48].

#### 3.2.9 Karanja oil

Karanja methyl ester produced by transesterification process. Karanja seeds contain 31% oil and 97% methyl ester was derived from Karanja oil [49]. The free fatty acids (FFA) of karanja oil is approximately 20% [50]. Annually biofuel can be produced 0.52 million tons from Karanja seeds by using unused land in Bangladesh [51].

#### 3.2.10 Castor oil

Castor is cultivated almost everywhere in the country. Stony, sandy and saline lands are most suitable for growing Castor. Castor oil seeds contain 67.7% oil [52]. The free fatty acid (FFA) content and viscosity have been found considerably higher such as 33.5% and 253 mm^2^/s, respectively [53].

#### 3.2.11 Neem

Among the many non-edible sources, Neem is one that is mainly cultivated in the rural areas. The oil content of Neem seeds is about 45% [23].

#### 3.2.12 Bahera

A huge amount of Bahera fruits is found in Bangladesh. Especially it is widely used as a medicinal treatment plant. Bahera contains about 30% oil by dry weight of crushed kernel [23].

#### 3.2.13 Rice bran oil (RBO)

The amount of rice husk generation in the year of 2010–11 was 6.71 million metric tons from 33.54 million metric tons of Paddy. Oil contains of rice husk is approximately 16–20% of its weight. Annual production of crude rice bran oil 1.34 million metric tons. These can be fulfilled 60%–70% of fossil fuel demand. The highest production rate was obtained with 0.9 % (wt. % of oil) catalyst use [54].

#### 3.2.14 Pitraj (aphanamixis polystachya)

Pitraj seeds can be a great source for biodiesel generation in our country. These seeds contain about 33.3% oil (wt/wt) [55]. Pitraj seeds also yield 79% oil [29].

### 3.3 Potentiality of other biodiesel feedstocks in Bangladesh

#### 3.3.1 Algae

The cultivation of algae is suitable in Bangladesh and it can produce in arid lands, waste water, clean water and saline water. At present, Bangladesh has 0.73 million hectares of unused land that's not suitable for cultivating any crops, which can be utilized for algae cultivation. Biodiesel can produce from lipids in the algae [9, 33]. Table 3 shows that lipid extraction from various microalgae, method of application, and yield of algal oil. The number of algae species varies from 30,000 to over 1 million. Some of algae species contain up to 80% lipid oil of their dry weight. The production of 1kg of Algae Biodiesel needs 1.83 kg of CO_2_ [23].

**Table 3:** Lipid extraction from algae [56].

Microalgae	Method of lipid extraction	Yield of algal oil
*Chlorella vulgaris*/Cyanobacteria leptolyngbya	Ultrasonification (750 W)	16.90%
Scenesdemus quadricauda	Microwave 600W	49%
Chlorella sp.	Parameciumbursaria Chlorella virus 1 (PBCV-1)	C18:3; 10.84 0.60
Chlorophyta sp.	Soxhlet extraction	18.29 0.4 wt%

### 3.4 Summary of biodiesel production in Bangladesh

Different types of feedstocks are studied here to observe their potentiality of biodiesel and bioethanol production in Bangladesh. Table 4 demonstrates the important findings from feedstocks in Bangladesh for biodiesel production. Currently, edible and non-edible together produce most of the biodiesel on the commercial scale. In Bangladesh has a good number of edible feedstocks are available. To achieve it, at first, all most of the edible come from soybean, mustard, coconut, sesame, and Mosna oil (Table 4). Sesame contains 42%–50% oil that's comparatively higher than the soybean and Mustard but the production capacity of soybean and Mustard is much higher in the country. Edible sources can meet the requirement of raw materials for biodiesel production, but these also used as the food oil in the country. So it has not ability to fulfill the demand for biodiesel production from edible sources. Secondly, non-edible sources can meet the requirement of biodiesel in our country. Non-edible sources are most effective to perform in Bangladesh for biodiesel production. Rice bran oil, Rubber seed oil, Cottonseed oil, Jatropha oil, Castor oil, Karanja oil, Neem, Bahera, algae, and Pitraj, etc. are promising sources for non-edible oil in our country. Bangladesh has lots of arable lands, non-arable lands, marshy lands, saline lands, road and railway side areas, fresh water, saline water and waste water for cultivation of non-edible biodiesel feedstocks and can easily meet the demand of biodiesel in our country. The highest biodiesel production capacity of rice brand oil is 0.9 % (wt. % of oil) and Oil contains of Algae about 80% of its dry weight.

**Table 4:** Summary of biodiesel production in Bangladesh.

Sources	Feedstock	Reference	Major Findings
Edible	Soybean oil	[30, 31, 32]	1) Soybean could be produced 2.25–2.4 metric tons per hectare in every year. 2) Approximately 7–8 lakh hectares of land could be used under Soybean cultivation. 3) Soybean could be produced 17 to 18 lakh metric tons per annum. 4) Soybean yield is about 20% oil
	Mustard oil	[9, 23, 33, 34]	1) Annual production of Mustard oil 22 lakh metric tons. 2) Mustard seeds can be a vital source of biodiesel. 3) The calorific value of mustard biodiesel fuel is 39.51 MJ/kg, which is same to the conventional fossil fuel, but the generation cost is slightly higher 4) Oil contains of mustard seeds is 41.5%.
	Coconut oil	[39, 40]	1) In 2004 to 2005, Bangladesh produced about 907255 metric tons of coconut by utilizing 12825 acre of land. 2) The properties of coconut oil compared to biodiesel satisfied with ASTM standards.
	Sesame oil	[37, 38]	1) Annual sesame production 25000 metric tons from 96000 hectares of arable land in Bangladesh. 2) Sesame contains 42%–50% oil.
	Mosna oil	[9, 35]	1) Mosna is mainly cultivated in Barisal, Comilla, and Chittagong, which are located in the southern part of Bangladesh. 2) The generation cost per liter of biodiesel from Mosna oil is Tk. 285.
Non Edible	Rice bran oil	[54]	1) The amount of rice husk generation in the year of 2010–11 was 6.71 million metric tons from 33.54 million metric tons of Paddy. 2) Oil contains of rice husk is approximately 16–20% of its weight 3) Annual production of crude rice bran oil 1.34 million metric tons. 4) The highest biodiesel production capacity is 0.9 % (wt. % of oil).
	Rubber seed oil	[41, 42, 43, 44]	1) The rubber seed yield is about 49% oil. 2) Annually extraction of Rubber seed oil per hectare is 217 kg. 3) Bangladesh can be used 91.8 thousand hectares of land for rubber seed cultivation which is14.7% of the total planted forest area. 4) It is possible to produce 0.04 million MT of biodiesel from rubber seed oil in every year.
	Cottonseed oil	[23, 35, 36]	1) Cottonseed oil can be used as massive sources of biodiesel production in Bangladesh. 2) Characteristics of the cottonseed oil are similar to conventional diesel fuel. 3) Cottonseeds produce approximately 77% Biodiesel with 20% Methanol.
	Jatropha oil	[45, 46]	1) *Jatropha curcas* seeds contain approximately 30–45% oil. 2) Free fatty acids (FFA) of Jatropha oil are about 14.02%.
	Castor oil	[52, 53]	1) The oil content of Castor seeds is about 67.7%. 2) The free fatty acid (FFA) content of Castor seeds is 33.5%.
	Karanja oil	[49, 51]	1) The amount of oil contains Karanja seeds is 31%. 2) Karanja oil could be produced 97% methyl ester which is relatively high. 3) Annually biofuel can be produced 0.52 million tons from Karanja seeds by using unused land in Bangladesh.
	Neem	[23]	1) The amount of oil content of Neem seeds is 45%. 2) Neem seeds can be used as a great sources to produce diesel fuel.
	Bahera	[23]	1) The huge amount of Bahera can be produced in Bangladesh By using unused land. 2) The amount of oil content of Bahera fruits about 30%.
	Pitraj	[17]	1) The amount of oil content of Pitraj oil about 33.3% (wt/wt).
Others	Algae	[9, 23, 33]	1) Oil content of Algae about 80% of its dry weight. 2) Approximately 0.73 million hector unused land can be used for Algae production. 3) The production of 1kg of Algae Biodiesel needs 1.83 kg of CO_2_.

From all the observations, it is found that the biodiesel generation capacity can be increased if edible and non-edible feedstocks are cultivated with the proper technology. In the context of Bangladesh, Soybean, Mustard, Algae can give the maximum effort for raising the biodiesel production capability. In addition, approximately 0.73 million hector unused land can be used for Algae production. Moreover, annual production of Soybean and Mustard oil is 18 and 22 lakh metric tons, respectively.

## 4 Bioethanol

Bioethanol is one of the great and environmentally friendly suitable sources of biofuels [57]. Bioethanol is produced by fermentation, especially from carbohydrates made in starch crops or sugar, such as potato, corn, sweet potatoes, sugarcane, Sugar beet, and sweet sorghum etc. Cellulosic biomass comes from non-food sources, like as grasses, and trees. Bioethanol can be used as a transportation fuel in its pure form, but it is blended to increase gasoline additive octane and vehicle emissions. USA and Brazil are the largest users of bioethanol. Table 5 shows the feedstocks for bioethanol production.

**Table 5:** Feedstocks for bioethanol production [57].

Starch sources	Sugar sources	Lignocellulosic sources
Corn (*Zea mays*)	Sugarcane (*Saccharum officinarum*)	Perennial grasses
Wheat (*Triticum aestivum*)	Sweet sorghum (*Sorghum bicolor*)	Aquatic plants
Cassava (*Manihot esculenta*)	Sugar beet (*Beta vulgaris*)	Agricultural residues
Barley (*Hordeum vulgare*)	Watermelon (*Citrullus lanatus*)	Forest biomass and waste
Canna (*Canna edulis*)	Dates (*Phoenix dactylifera*)	Municipal solid waste
Sorghum (grain) (*Sorghum bicolor*)	Molasses	
Rice (*Oryza sativa*)		
Sweet potato (*Ipomoea batatas*)		
Potato (*Solanum tuberosum*)		
Yam (*Dioscorea rotundata*)		
Jerusalem artichoke (*Helianthus tuberosus*)		
Iles-iles (*Amorphophalus campanulatus*)		
Oat (*Avena sativa*)		
Banana (Musa sp.)		

**Figure 2:**
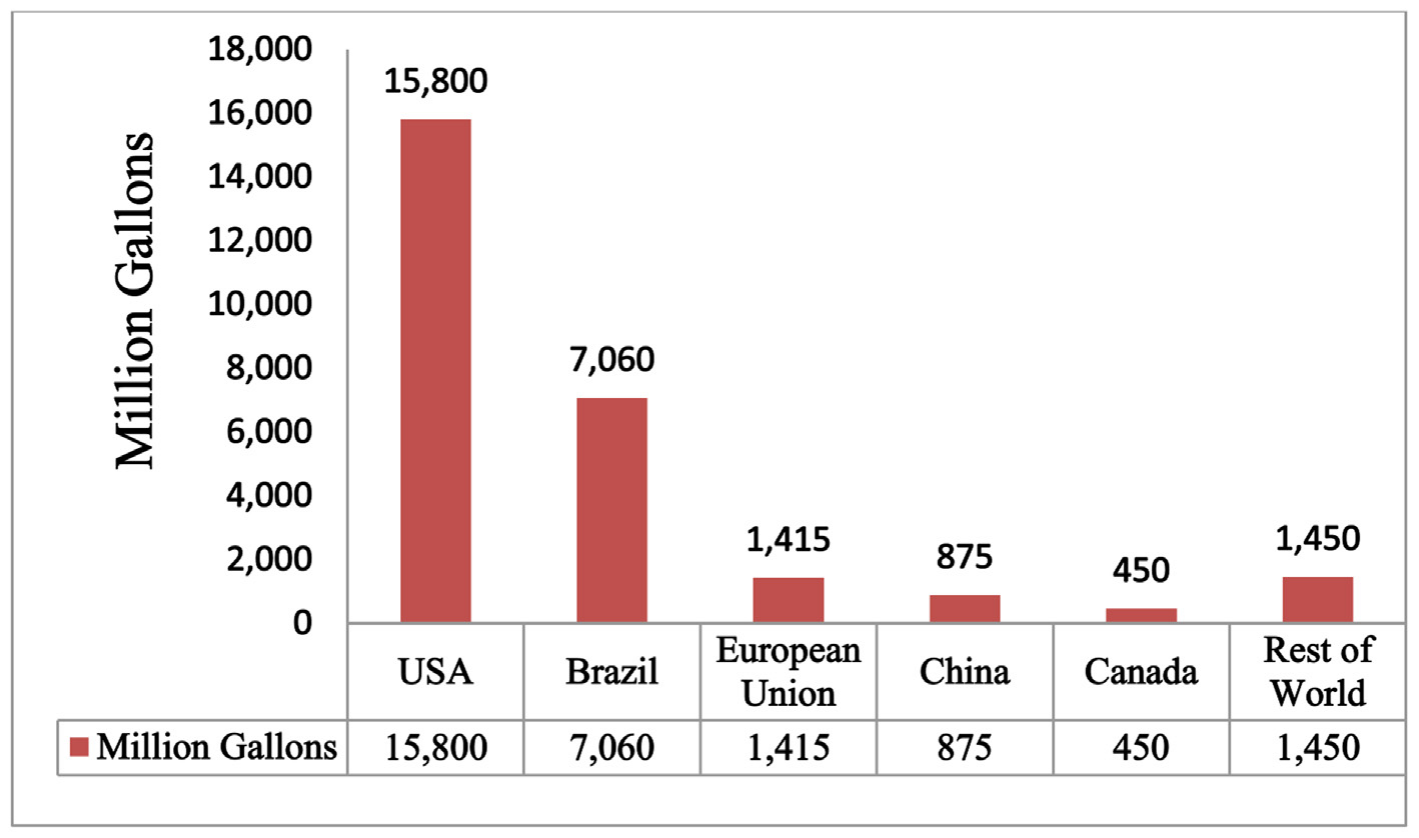
Global ethanol generation by Country or Region in 2017 [59].

Global ethanol generation capacity in 2005 and 2006 was about 45,420 million liters and 49,000 million liters, respectively [58]. Worldwide ethanol generation reached 73,900 million liters in 2009. Figure 2 shows that In 2017 worldwide production of ethanol fuel reached 27,050 Million gallons. United State and Brazil are the largest ethanol producer countries in the world and the fiscal year 2017 they produced 15,800 and 7,060 million gallons of ethanol fuels, respectively in the fiscal year 2017 [59]. Many countries used different feedstock for bioethanol production such as China and Canada are depends starchy sources like corn, rice, wheat and cassava, while Germany, Australia, France and India are depends sugary sources like sugarcane, wheat, sugar beet and molasses.

### 4.1 Fuel properties: bioethanol and bioethanol blend fuels

Table 6 contains key properties of petrol, octane and bioethanol. Bioethanol has a lower calorific value than that of octane and petrol. In general, energy content as 1 gallon of octane or petrol is equal to the 1.5 gallons of bioethanol from Table 6. As a result, bioethanol has needed more amount of contribute to the same function than either octane or petrol. On the other hand, flash point of bioethanol is higher than petrol and octane which is suitable in our country [60].

**Table 6:** Fuel properties of petrol, octane and bioethanol [61, 62].

Parameter	Unit	Petrol	Octane	Bioethanol
Lower heating value	MJ/kg	42.7	43.5	26.8
Flashpoint	°C	–7.2	–8	17.2
Density	kg/L	0.74	0.755	0.79
Reid vapor pressure	psi	7	8	2
Viscosity	cSt	0.64	0.645	1.2
Octane number	–	80	95	110

In fiscal year 2015–16, the consumption of transportation fuel was 137,000 metric tons of petrol and 147,000 metric tons of octane in Bangladesh [60]. Bioethanol made by fermentation of sugars, starch or lingo-cellulosic raw materials can be blended with petrol or octane in different amount of ratios. The government of Bangladesh is allowing a 5% blend of bioethanol with octane, petrol or gasoline [15]. Table 7 Contain petrol-bioethanol and octane-bioethanol blends 5, 10, 15 and 20% bioethanol are referred to as E5, E10, E15 and E20 respectively in this article.

**Table 7:** Fuel properties of bioethanol blends [61].

Parameter	Petrol and petrol-bioethanol blends				Octane and octane-bioethanol blends			
	E5	E10	E15	E20	E5	E10	E15	E20
Density (kg/L)	0.75	0.76	0.76	0.76	0.75	0.76	0.76	0.76
Reid vapor pressure (psi) @ 37.8 °C	6.75	6.8	6.35	6.1	6.95	7.2	6.9	6.85
Viscosity (cSt)	0.655	0.68	0.69	0.71	0.655	0.69	0.7	0.71
Octane number	82	83	85	86	96	97	97	98

### 4.2 Potentiality of different bioethanol feedstocks in Bangladesh

#### 4.2.1 Bioethanol production from agricultural residues

Agricultural residues are a great sources of bioethanol production. Bangladesh has available land for cultivation of crops like as corn, groundnut, wheat, maize, sugarcane, wood, rice, cotton, pulse, jute, and annually about 65.36 metric tons of agricultural crop residues are produced from the major crops. In 2010–2011, amount of agricultural residues in Bangladesh is approximately 58.503 million tones. Bangladesh can generate 32 metric tons bioethanol per annum by utilizing these crop residues [63]. In fiscal year 2019–2020, Bangladesh can produce about 31.65 million metric tons of bioethanol from rice residue [43, 64]. Table 8 shows the biomass and energy generation potential from different residues in Bangladesh.

**Table 8:** Potentiality of biomass and energy generation in Bangladesh [65].

Sources of Biomass	Biomass production (Million Tons)	Energy (PJ)	Electricity Generation (TWh)
Agricultural residues	94.1	582.68	161.8
Forest residues	17.44	210.74	58.53
Livestock residues	88.89	456.65	126.81
Municipal solid waste	13.38	95.55	26.57
Total	213.81	1345.62	373.71

#### 4.2.2 Bioethanol production from forest residues

Forest Residues are vital sources of biomass and 16.7% of land is used for forest in Bangladesh [66]. The organization of the UN Food and Agronomy studied that Bangladesh utilized 14,42,000 hectors of land for forest where 436,000 hectares are used for primary reserve forest with 237,000 hectors of artificial man-made forest. Biomass generation from wood per annum in Bangladesh is 167.4 million cubic feet where 5.2 million tons of fuel woods, and 1.0 million tons of bamboos [67].

#### 4.2.3 Bioethanol production from municipal and industrial solid wastes

**Figure 3:**
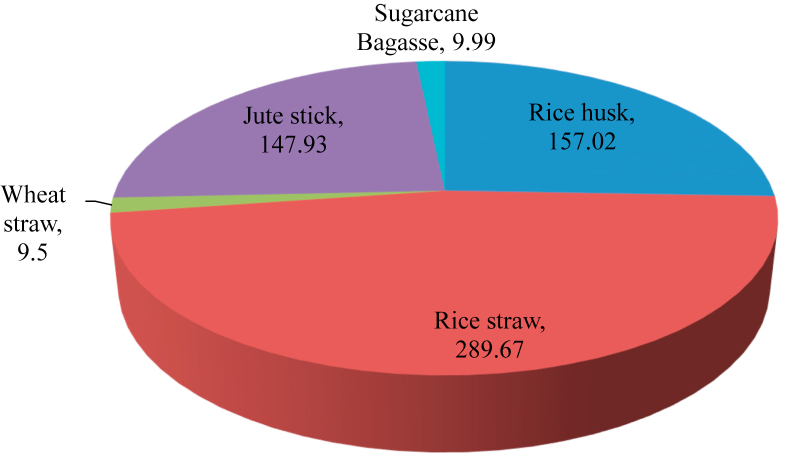
Energy potential in petajoule (PJ) for agricultural residue in Bangladesh in 2020 [69].

Municipal and Industrial Solid wastes are a vital source for biomass generation and energy production. Annually, the amount of waste produced in Bangladesh is about 22.4 million tons. In 2015, Dhaka South and North City Corporation are generated 3300 tons and 2800 tons of wastes per day were 20% of the waste came from biomedical waste. Bangladesh can earn from solid e-waste approximately 268240469 taka per annum [66, 68]. Figure 3 shows that annual biomass and waste generation statistical energy potentiality in Bangladesh.

#### 4.2.4 Bioethanol production from agricultural products

Bangladesh is agricultural country here produced many kinds of agricultural products and that can be used as a great sources of bioethanol production. For the experiment, each 100 g of boiled agricultural product (pumpkin, carrot, corn, and sweet potato) blend with 300 ml distilled water. To make a 500 ml solution also added 200 ml yeast with a blended solution for alcoholic fermentation, then incubated at 31 °C for 6-days. Bioethanol generation capacity of red pumpkin (*Cucurbita maxima* L.) and black color pumpkin are 53 ml and 40 ml of ethanol with the purity of 6 %v/v and 4 %v/v respectively. Carrot and Corn are produced 73.67 ml and 63.00 ml of ethanol with 12.66 % (v/v) and 13.33 % (v/v) purity [59]. In 2014, United States produced 14.3 billion gallons of ethanol from corn feedstock and exported approximately 825 million gallons of ethanol in different countries [70]. Sweet potato can produce the maximum amount of bioethanol with 35% purity [71]. Potatoes are important sources of bioethanol and a large amount of potatoes are produced in Bangladesh every year [72]. The total generation of potato surrplus was 3.22 million metric tons from 10.22 million metric tons of potatoes in 2017–18 fiscal years. Only by using the surplus can be produced 143670082.36 gallons of bioethanol that can easily meet the requirement for 5% blending of bioethanol annually [73].

#### 4.2.5 Bioethanol production from fruits

Different kinds of fruits are produced in Bangladesh such as orange, banana, papaya, sapota, Mango (*Mangifera indica* L.), and wild date palm which could be produced huge amount of bioethanol. The bioethanol generation capacity of local varieties of banana called Sagorkola (*Musa sapientum* L.) is considerably higher than orange, papaya and sapota fruits. The purity of bioethanol in a single distillation of banana was 40% [71]. Mango (*Mangifera indica* L.) is a promising source of bioethanol generation. The worldwide production of mango in 2017 and 2019 are 47.13 million tons and 1047850 tons respectively [74]. In Bangladesh have local varieties of mango such as Lengra, Khershapat, Amropali, Fazli and Lakhna. Khershapat (*Mangifera indica* L.) contain the highest amount of bioethanol, which approximately 77.67 g/L with 32% (v/v) purity [75]. Wild date palm is another source of bioethanol production. It is a highly sugary feedstock and minimum 8076.62 L of ethanol could be produced from an orchard comprising 500 plants per hectare [76].

### 4.3 Summary of bioethanol production in Bangladesh

Table 9 shows the summary of bioethanol production in Bangladesh. Bioethanol produced from various sources of feedstocks such as starchy, sugary, lignocellulosic. To observe it, at first, from starchy sources, Sagorkola banana and Sweet potato contain high amount of bioethanol with 40% and 35% purity, respectively. The maximum amount of bioethanol generation capacity of Sweet sorghum is 9%, w/v from Sugary sources. On the other hand, only agricultural residues can meet the demand for bioethanol production.

**Table 9:** Summary of bioethanol production in Bangladesh.

Sources	Feedstocks	Reference	Condition	Major findings
Starchy	Corn	[57, 59, 70]	Each 500 ml solution.	1) Corn yields 83.38% of ethanol. 2) Corn produced 63.00 ml of ethanol with the purity of 13.33 % (v/v). 3) In 2014, United States produced 14.3 billion gallons of ethanol from corn feedstock and exported approximately 825 million gallons of ethanol in different countries.
	Sweet potatoes	[71]	Each 500 ml solution.	1) The sugar and bioethanol content in sweet potato was 13.96 mmol/L and (95 ml) with 35 % (v/v) purity respectively. 2) A huge amount of sweet potato is produced in Bangladesh.
	Potato	[71, 72, 73]	Each 500 ml potato starch solution.	1) Bangladesh can produce 143670082.36 gallons of bioethanol by using unused portion and surplus of potato that's enough to meet the demand of 5% bioethanol blend annually. 2) In the fiscal years 2017–18, Bangladesh was produced 10.22 million metric tons of potato. 3) The purity of ethanol was 10%–12% (v/v).
	Pumpkin	[59]	Each 500 ml solution.	1) Red pumpkin and black color pumpkin produce 53 ml and 40 ml of bioethanol with the purity of 6 %v/v and 4 %v/v respectively. 2) Available Agri-product in Bangladesh.
	Carrot must	[57, 59]	Each 500 ml solution.	1) Carrot produces 73.67 ml of bioethanol with purity 12.66 % v/v. 2) Ethanol production from the carrot must is about 77.5 L/ton under the optimum conditions.
	Banana	[71]	Each 500 ml solution.	1) Four different varieties of banana sagor, sabri, champa and bitchikola are producing 97 ml, 87 ml, 83 ml, and 81 ml of bioethanol with purity 40% v/v, 30% v/v, 35% v/v, and 20% v/v respectively. 2) Sagorkola banana contains high amount of bioethanol with 40% purity.
	Mango	[75]	Each 500 ml mango pulps solution.	1) Five different varieties of mango Lengra, Khershapat, Amropali, Fazli and Lakhna are producing 65 ml, 77.67 ml, 76.33 ml, 64.33 ml and 57.67 ml of bioethanol with purity 28% v/v, 32% v/v, 29% v/v, 27.67% v/v and 26% v/v respectively. 2) In the world, Bangladesh is the largest mango producing country.
	Wheat	[57]	–	1) Efficiency of ethanol from wheat starch is nearly 95%.
	Rice wine cake	[57]	–	1) Efficiency of ethanol after fermentation was found 94.0%.
Sugary	Sugarcane	[57]	–	1) Worldwide annual production of sugarcane about 360 million tons. 2) Sugarcane crop contains 60–79.5 t/ha.
	Sweet sorghum	[57]	–	1) Bangladesh has lots of arid land that can be used for cultivation of Sweet sorghum. 2) The maximum amount of bioethanol generation capacity is 9%, w/v.
	Dates	[57]	–	1) Ethanol production from Kunta, Eguoua and Bouhatem around 25% (v/v).
	Sugar beet	[57]	–	1) Sugar beet yield about 79.1 t/ha. 2) Ethanol yield per ton of stalk is approximately 95 L.
Lignocellulosic	Agricultural residues	[63]	–	1) Bangladesh can generate 32 Mt bioethanol fuels by utilizing agricultural residues. 2) The amount of agricultural residues in Bangladesh is nearby 65.36 metric tons per annum.
	Municipal solid waste	[66, 68]	–	1) Annually, the amount of waste produced in Bangladesh is about 22.4 million tons 2) Bangladesh can earn from solid e-waste approximately 268240469 taka per annum.
	Forest residues	[65, 66]	–	1) The volume of wood fuel in Bangladesh about 27662000 m^3^. 2) Annual biomass generation of forest residues about 17.44 million tons.

To summarize the observations, the findings were coming from various feedstocks for bioethanol production. Maximum studies are related to improvement and outcomes of sustainable feedstocks for bioethanol production in Bangladesh. Bioethanol production capacity will be increased by utilizing the different types of feedstocks like as an agricultural product, residues and waste. Agricultural product and residues are more efficient for increasing the bioethanol production capability in the country. Bangladesh can generate 32 metric tons bioethanol fuels by utilizing agricultural residues.

## 5 Future prospects

Bangladesh is an agricultural based country. There has lots of waste and unused land for cultivating biodiesel and bioethanol production sources. Bangladesh government can install industrial plant to generate biofuel in commercial scale for proper utilization of biodiesel and bioethanol production sources. As a result, dependency on the fossil fuel will be reduced in our country and also play the important role in the reduction of greenhouse gas emissions.

## 6 Conclusion

The climate of Bangladesh is suitable for biofuel production. Some edible, non edible, starchy, sugary, lignocelluloses sources can be the suitable candidates for biodiesel and bioethanol production in Bangladesh. The paper reveals the potentiality of various biofuel feedstocks and sources like as soybean oil, mustard oil, cottonseed oil, sesame oil, coconut oil, algae, rubber seed oil, jatropha, karanja oil, castor, bahera, neem, rice bran oil, pitraj and also different types of residues, crops, fruits, wastes. The following conclusions can be drawn.

(a) Bangladesh has the ability to produce annually about 0.16 million tons of edible oil. In addition, about 1001881 tons of biodiesel can be produced from 23,87,500 tons of non-edible oil.
(b) Approximately 32 metric tons of bioethanol from 65.36 metric tons of agricultural crop residues, and about 143670082.36 gallons of bioethanol from 10.22 million metric tons of potato that is enough to meet the demand of 5% bioethanol blend annually in Bangladesh.
(c) Approximately 0.04 million metric tons of biodiesel can be made from rubber seed oil and 1.34 million metric tons of crude rice bran oil from rice husk of the paddy.

It is conjectured that these annual production of various feedstocks can be used as the major sources of biofuel and also can meet the demands of biofuel in Bangladesh.

## Declarations

### Author contribution statement

All authors listed have significantly contributed to the development and the writing of this article.

### Funding statement

This research did not receive any specific grant from funding agencies in the public, commercial, or not-for-profit sectors.

### Data availability statement

Data will be made available on request.

### Declaration of interest’s statement

The authors declare no conflict of interest.

### Additional information

No additional information is available for this paper.
